# Efficacy and safety of Cadonilimab in the treatment of recurrent/metastatic and advanced cervical cancer: a systematic review and meta-analysis

**DOI:** 10.3389/fimmu.2025.1729380

**Published:** 2026-01-12

**Authors:** Qiuqi Zhuang, Yan Liu

**Affiliations:** Fujian University of Traditional Chinese Medicine, Fuzhou, China

**Keywords:** Cadonilimab, cervical cancer, efficacy and safety, recurrent and metastatic, systematic review and meta-analysis

## Abstract

**Objective:**

To investigate the efficacy and safety of Cadonilimab in patients with metastatic, recurrent, and advanced cervical cancer.

**Methods:**

Computerized searches were performed in PubMed, Embase, Cochrane Library, Wiley Online Library, Web of Science, and the CBM database from their inception until October 4, 2025, to collect published studies on Cadonilimab for cervical cancer. A single-arm rate meta-analysis was conducted using R software (version 4.4.1) to evaluate outcome measures including efficacy, safety, and prognostic indicators.

**Results:**

Ten studies involving 728 patients were included. The meta-analysis yielded the following pooled results: the overall response rate (ORR) was 57.8% (95% CI = 44.5%-70.2%), the disease control rate (DCR) was 81.8% (95% CI = 69.5%-89.9%), the complete response (CR) rate was 16.1% (95% CI = 10.2%-24.4%), and the partial response (PR) rate was 37.4% (95% CI = 28.6%-47.2%). The rates of stable disease (SD) and progressive disease (PD) were 19.8% (95% CI = 13.6%-27.9%) and 18.2% (95% CI = 10.1%-30.5%), respectively. The 6-month, 12-month, and 24-month overall survival (OS) rates were 78%, 78%, and 62%, respectively, while the 6-month and 12-month progression-free survival (PFS) rates were 53% and 48%, respectively. Regarding safety, the incidence of any-grade treatment-related adverse events (TRAEs) was 96.4% (95% CI = 75.5%-99.6%), the rate of grade ≥3 adverse events was 41.1% (95% CI = 25.2%-59.1%), and immune-related adverse events (irAEs) occurred in 38.6% (95% CI = 23.0%-56.9%) of patients.

**Conclusion:**

This meta-analysis suggests that Cadonilimab is associated with antitumor activity and a manageable safety profile in patients with recurrent, metastatic, and advanced cervical cancer. Close monitoring for irAEs, vigilance against adverse events, and timely pharmacological intervention are essential during treatment to mitigate risks and achieve optimal therapeutic outcomes.

**Systematic Review Registration:**

https://www.crd.york.ac.uk/PROSPERO, identifier CRD420251168672.

## 1 Introduction

Globally, CC has become the fourth most common cancer and the fourth leading cause of cancer death among women, imposing a substantial socioeconomic burden, and the global burden is projected to increase significantly by 2030 (1, 2). Chronic high-risk human papillomavirus (HR-HPV) infection leads to cervical disease, with infected women having a 75.4-fold higher risk of developing CC compared to HPV-negative women. HPV-16 and HPV-18 have been established as the most oncogenic genotypes, accounting for approximately 70% of CC cases worldwide and representing the primary causative agents (3, 4). Early-stage CC is often difficult to recognize due to the absence of symptoms. As the disease progresses, the most common clinical manifestations include irregular or heavy vaginal bleeding, atypical blood-stained vaginal discharge, and increased vaginal discharge (5). The 5-year progression-free survival and overall survival rates for patients with locally advanced CC are approximately 50% to 80%, yet around 30% of patients still face disease recurrence (6, 7). Patients who experience recurrence after prior standard therapy or are diagnosed with metastatic disease have significantly diminished survival rates, with a 5-year survival rate of merely 10% (6). Standard treatment options for CC typically involve surgical intervention for early-stage disease and concurrent chemoradiotherapy for locally advanced disease. Although historically, treatment options were limited and prognosis remained poor with high mortality rates for patients with disease progression after first-line platinum-based chemotherapy (5, 8), in recent years, immune checkpoint inhibitors combined with chemotherapy have become the standard first-line treatment recommended by the NCCN guidelines. However, for this patient population, there remains room for improvement in efficacy, underscoring the urgent need for more effective therapeutic options. Therefore, novel therapies are urgently required to address this challenge.

With immune checkpoint inhibitors (ICIs) demonstrating encouraging efficacy in the treatment of CC, ICIs have now been approved for use in both first-line and second-line CC therapy (9), Immune checkpoint therapy functions by blocking inhibitory signals on T cells, thereby activating their anti-tumor immune function. CTLA-4, an inhibitory checkpoint molecule, primarily regulates T-cell activation during the initial phase, while the PD-1/PD-L1 pathway suppresses T-cell effector functions within the tumor microenvironment (10). Cadonilimab, a PD-1/CTLA-4 bispecific antibody, has been applied in the treatment of various solid tumors including CC, lung cancer, gastric/gastroesophageal junction cancer, esophageal squamous cell carcinoma, hepatocellular carcinoma, and nasopharyngeal carcinoma (11). Furthermore, it received initial approval in China in June 2022 for the treatment of patients with recurrent or metastatic CC who experienced disease progression during or following platinum-based chemotherapy (11).

Cadonilimab, as a novel bispecific antibody, has demonstrated its therapeutic potential in small-sample, early-phase clinical trials, which was ultimately confirmed in the pivotal phase III trial (COMPASSION-16). However, efficacy outcomes vary significantly across different settings, and a comprehensive synthesis of the evidence is lacking. The systematic integration and comparison of efficacy data from clinical studies of different phases and designs remain of significant value for a thorough understanding of the drug’s benefit-risk profile. Furthermore, in clinical practice, combination therapy with anti-CTLA-4 and anti-PD-1 antibodies appears to increase the incidence of all-grade adverse events and immune-related adverse events (12, 13). Consequently, the balance between safety and efficacy remains unclear, and standardized risk assessment criteria are lacking. Therefore, we conducted this meta-analysis, synthesizing the latest data from 10 studies involving 728 patients—including both real-world studies and clinical trials—to comprehensively evaluate the efficacy and safety of Cadonilimab in patients with recurrent/metastatic and advanced CC, thereby providing evidence-based medical support for precise clinical medication.

## 2 Materials and methods

### 2.1 Search strategy

We systematically searched electronic databases including PubMed, Embase, the Cochrane Library, Wiley Online Library, Web of Science, and CBM from their inception until October 4, 2025. Our search was global in scope, with no restrictions on language or region. The search strategy employed a combination of subject headings and free-text terms, which were adjusted according to each database’s specific features. We aimed to identify published literature such as cohort studies, case-control studies, randomized controlled trials, and clinical trials investigating Cadonilimab in patients with recurrent/metastatic or advanced CC. English search terms included “Uterine Cervical Neoplasm”, “Cadonilimab”, among others. The specific search strategy used for PubMed is provided in **Supplementary Content 1**.

### 2.2 Study selection

Studies meeting the following criteria were included in this meta-analysis: (a) patients with a confirmed diagnosis of recurrent, metastatic, or advanced CC, regardless of age, region, ethnicity, or prior treatment history; (b) patients receiving Cadonilimab infusion therapy, either alone or in combination with foundational treatments such as radiotherapy, chemotherapy, or surgery; (c) studies reporting at least one of the following: efficacy, safety, or prognostic outcomes.

Studies were excluded if they met any of the following criteria: (a) tumor types other than CC; (b) full text unavailable or lacking usable statistical data; (c) publication types such as reviews, secondary analyses, case reports, conference abstracts, animal studies, or *in vitro* studies.

### 2.3 Outcome indicators

The outcomes primarily included efficacy, safety, and prognostic indicators. Efficacy indicators comprised the objective response rate (ORR), disease control rate (DCR), complete response rate (CR), partial response rate (PR), stable disease rate (SD), and progressive disease rate (PD). Safety indicators included treatment-related adverse events (TRAEs) of any grade, grade ≥3 adverse events, immune-related adverse events (irAEs), hematologic toxicity, hepatic toxicity, gastrointestinal toxicity, and endocrine toxicity. Prognostic indicators included overall survival (OS) and progression-free survival (PFS). The primary outcome measures of this study were progression-free survival (PFS) and overall survival (OS). Secondary outcome measures included efficacy endpoints and safety indicators. Although the synthesis of OS/PFS was a primary analytical goal, the pooled estimates are derived from a limited number of studies and must therefore be approached with considerable caution.

### 2.4 Data screening and extraction

Two researchers independently performed literature screening and data extraction using EndNote X9 for reference management. The process involved removing duplicate records using the software, excluding clearly irrelevant studies after a preliminary review of titles and abstracts, and applying inclusion and exclusion criteria for rigorous screening. Any discrepancies were resolved through discussion, with involvement of a senior researcher when necessary. Extracted data included first author, publication year, sample size, dosage regimen, mean age, and outcome measures. Attempts were made to contact corresponding authors for missing data.

### 2.5 Quality assessment

The risk of bias in included studies was independently assessed by two investigators. The MINORS scale was used for non-randomized studies, comprising 12 items: 8 applicable to non-comparative studies and 4 additional items for comparative studies. Each item was scored 0–2 points (0: not reported; 1: reported but inadequate; 2: adequately reported), yielding maximum scores of 16 and 24 points for non-comparative and comparative studies, respectively. Randomized controlled trials were evaluated using the Jadad scale (maximum 7 points), with scores of 1–3 indicating low quality and 4–7 indicating high quality.

### 2.6 Statistical analysis

This meta-analyses of single-arm proportions were conducted using R software (version 4.4.1). Treatment outcomes for CC patients receiving Cadonilimab were systematically evaluated, with endpoint measures expressed as event rates with 95% confidence intervals (CIs). Heterogeneity was assessed using Q-test and I² statistics, with P > 0.10 and I² < 50% indicating low heterogeneity, and P ≤ 0.10 or I² ≥ 50% indicating substantial heterogeneity. Given the variable heterogeneity across studies, all analyses employed random-effects models for pooled rates. Sensitivity analyses were performed to explore heterogeneity sources, while publication bias was assessed using funnel plots and Egger’s test. Statistical significance was defined as P < 0.05.

## 3 Results

### 3.1 Systematic search

**Figure 1:**
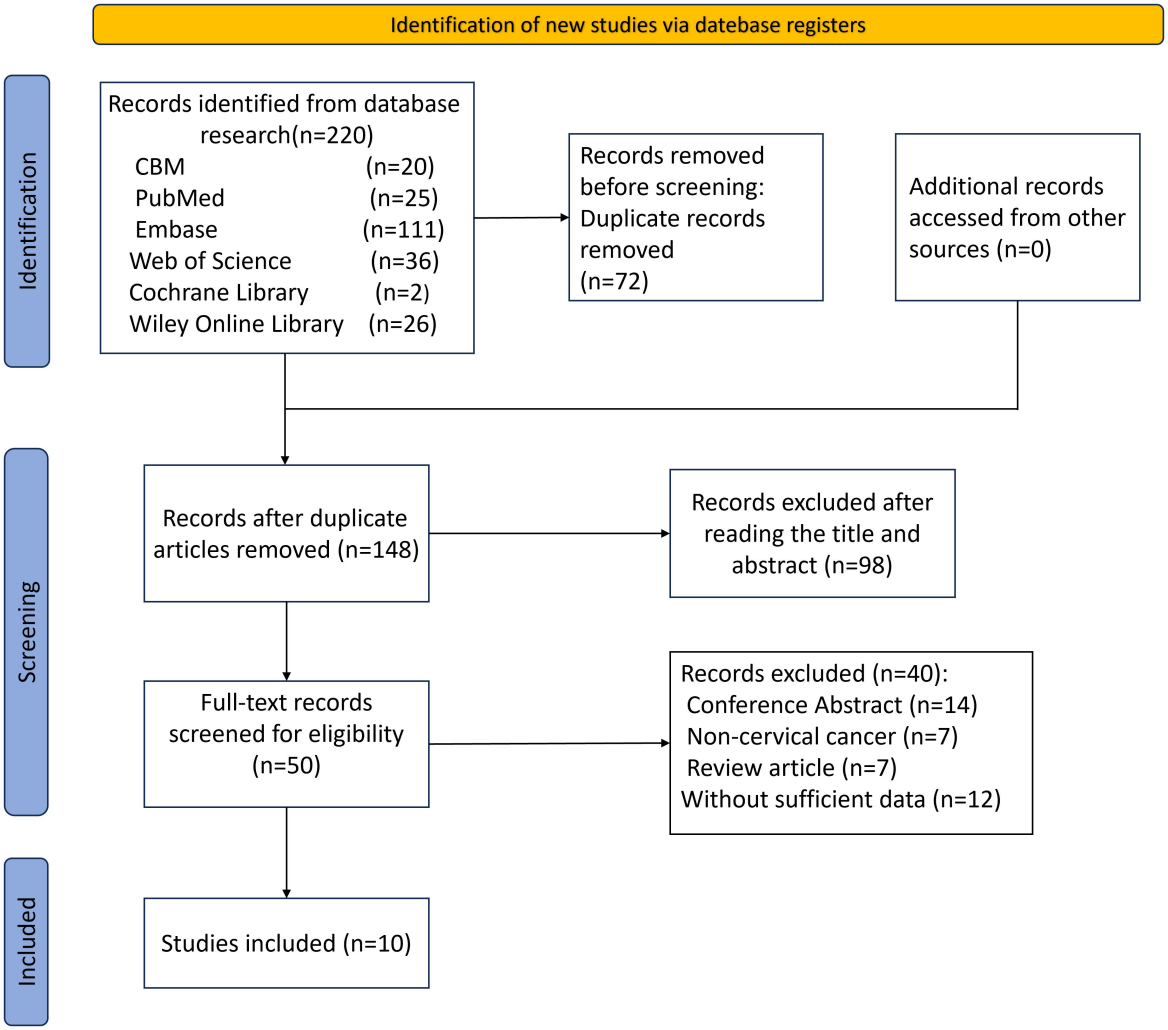
Flowchart of literature screening.

The initial database search identified 220 records. Through sequential screening of titles and abstracts, followed by rigorous application of the inclusion and exclusion criteria, 10 studies (14–23) were ultimately included. These comprised 7 retrospective cohort studies (14–19, 23) and 3 clinical trials (20–22), involving a total of 728 CC patients who received Cadonilimab treatment. These studies were all conducted by different investigators at separate, independent cancer hospitals or medical centers. The literature screening process is shown in **Figure 1**.

### 3.2 Study characteristic

The sample sizes across the 10 included studies ranged from 15 to 222, totaling 728 CC patients with ages ranging from 18 to 86 years. All studies evaluated treatment response using the WHO Response Evaluation Criteria in Solid Tumors (RECIST v1.1). The basic characteristics and quality assessment results of the included studies are presented in **Table 1**.

**Table 1:** Basic characteristics and quality evaluation of the literature.

No.	Study/Year	Region	Research scale	Study design	Cadonilimab dosage	Outcomes			Sample size	Age	Criteria	Score
						Efficacy	Safety	Prognosis				
1	Chen 2025	China	Multicenter	Retrospective cohort study	10mg/kg, q3w or 6mg/kg, q2w	①②③④⑤⑥	⑦⑧⑨⑩⑪⑫⑬	⑭⑮	139	53(26-86)	RECIST v1.1	11
2	Pan 2025	China	Single-center	Retrospective cohort study	10mg/kg, q3w	①②③④⑤⑥	⑦⑧⑨⑩⑪⑫⑬	—	101	53 (27-73)	RECIST v1.1	18
3	Ge 2025	China	Single-center	Retrospective cohort study	10mg/kg, q3w	①②③④⑤⑥	⑦⑧⑨⑩⑪⑬	—	25	50.92±11.15	RECIST v1.1	11
4	Wang 2025	China	Single-center	Retrospective cohort study	10mg/kg, q3w	①②③④⑤⑥	⑩⑪⑫⑬	⑭⑮	50	18-75	RECIST v1.1	18
5	Han 2025	China	Single-center	Retrospective cohort study	10mg/kg, q3w	①②③④⑤⑥	⑧⑨⑩⑬	⑭	21	57(28-74)	RECIST v1.1	11
6	Zhou 2025	China	Single-center	Retrospective cohort study	10mg/kg, q3w	①②③④⑤⑥	⑦⑧⑨⑩⑪⑬	—	19	58.5(42-74)	RECIST v1.1	11
7	Wu 2024	China	Multicenter	Random control trial	10mg/kg, q3w	①②③④⑤⑥	⑦⑧⑨⑩⑪⑫⑬	⑭	222	56 (23–75)	RECIST v1.1	7
8	Lou 2024	China	Single-center	Clinical trial	15mg/kg, q3w	①②③④⑤⑥	⑦⑧⑨⑩⑪⑫⑬	⑭⑮	15	52.4 (35–67)	RECIST v1.1	12
9	Gao 2023	China	Multicenter	Clinical trial	6mg/kg, q2w	①②③④⑤⑥	⑩⑪⑫⑬	⑭⑮	111	52 (45–58)	RECIST v1.1	14
10	Chunyan 2025	China	Single-center	Retrospective cohort study	10mg/kg, q3w	①②③④⑤⑥	⑧⑩⑫⑬	—	25	55.5±9.3	RECIST v1.1	12

①ORR ②DCR ③CR ④PR ⑤SD ⑥PD ⑦Any-grade TRAEs ⑧grade≥3 TRAEs ⑨irAEs ⑩hematologic toxicity ⑪hepatic toxicity ⑫gastrointestinal toxicity ⑬endocrine toxicity ⑭OS ⑮PFS.

### 3.3 Therapeutic efficacy assessments

**Figure 2:**
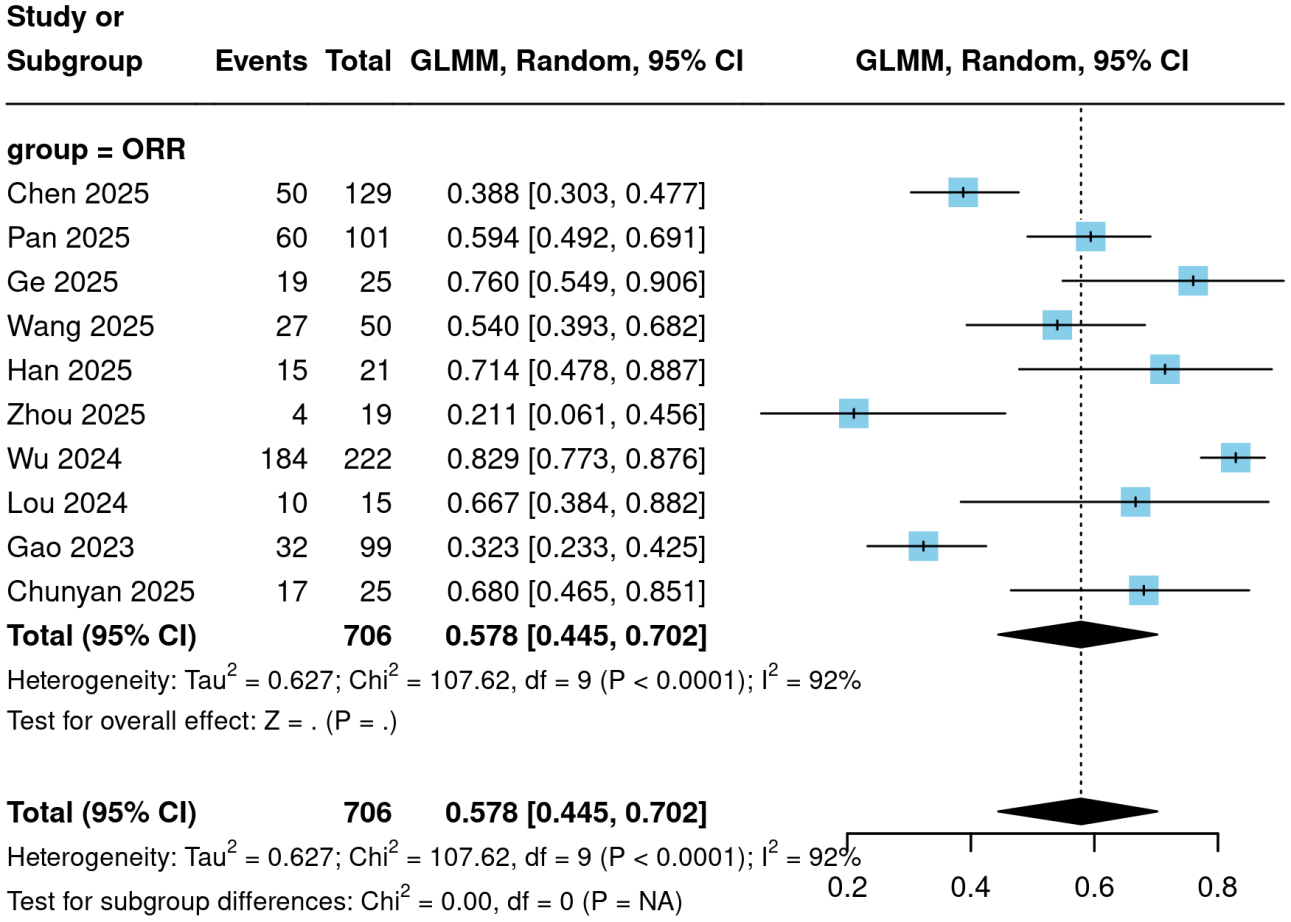
Forest plot of ORR.

**Figure 3:**
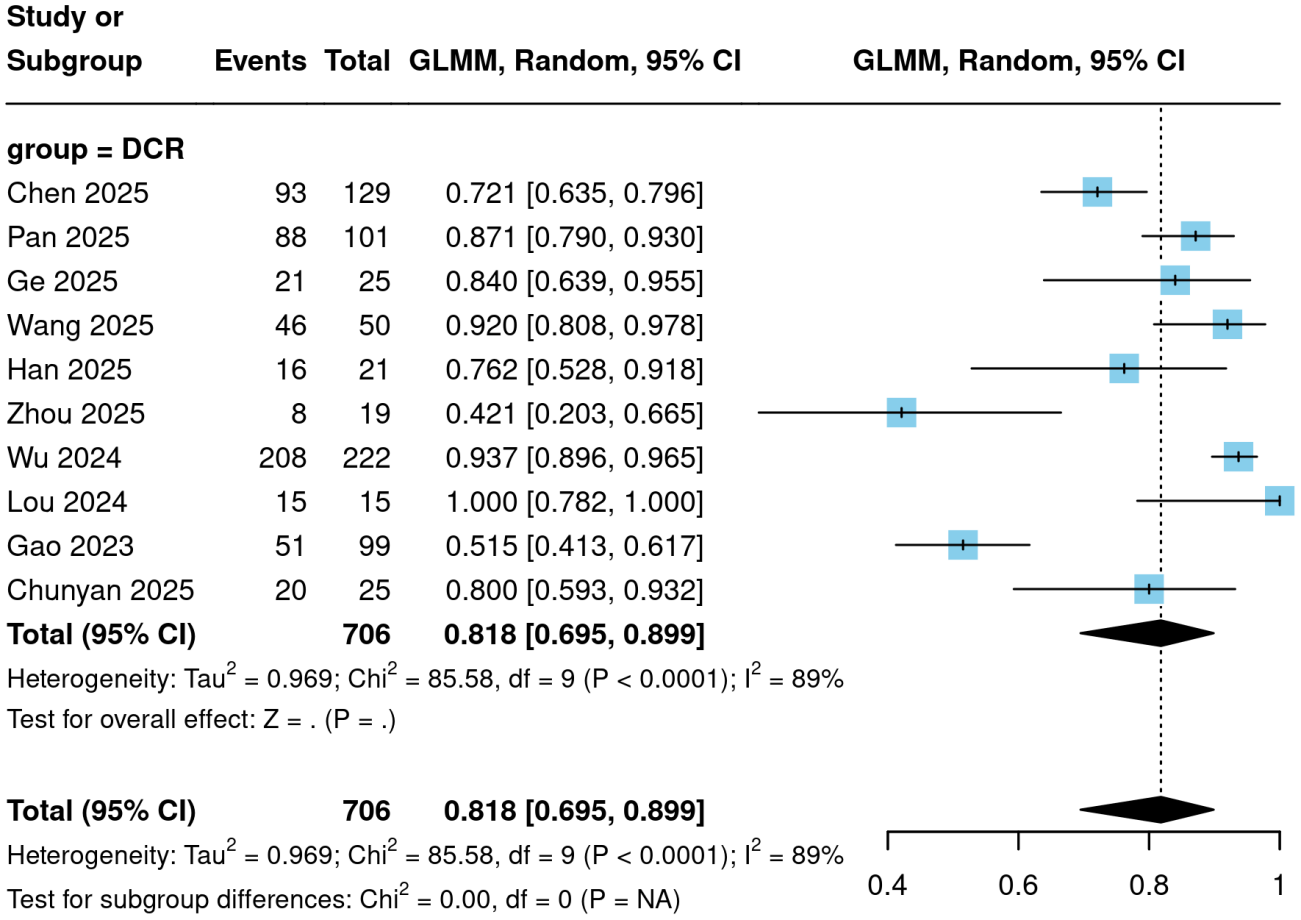
Forest plot of DCR.

**Figure 4:**
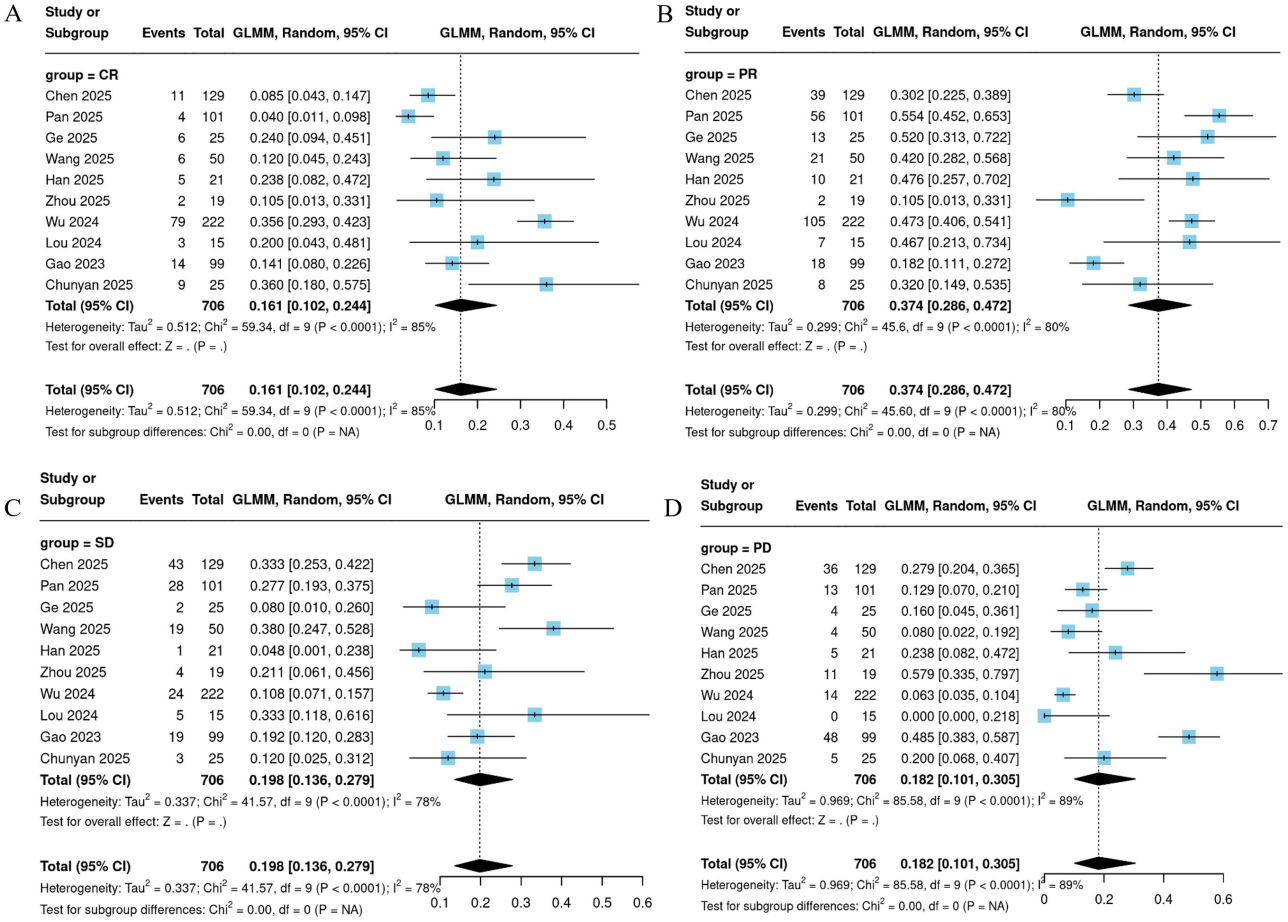
Forest plot of CR **(A)**, PR **(B)**, SD **(C)**, PD **(D)**.

All 10 included studies reported the six key efficacy endpoints of ORR, DCR, CR, PR, SD, and PD for CC patients treated with Cadonilimab. Meta-analysis results demonstrated that ORR ranged from 21.1% to 82.9%, with a pooled result of 57.8% (95% CI = 44.5%-70.2%; **Figure 2**). DCR ranged from 42.1% to 100%, with a pooled result of 81.8% (95% CI = 69.5%-89.9%; **Figure 3**). Among the 706 patients evaluated for efficacy, 139 achieved a complete response, yielding a pooled proportion of 16.1% (95% CI = 10.2%-24.4%); 279 patients achieved a partial response, with a pooled proportion of 37.4% (95% CI = 28.6%-47.2%); 148 patients had stable disease, accounting for 19.8% (95% CI = 13.6%-27.9%); and 140 patients experienced disease progression, representing 18.2% (95% CI = 10.1%-30.5%). These results are detailed in **Figure 4**.

### 3.4 Safety assessments

**Figure 5:**
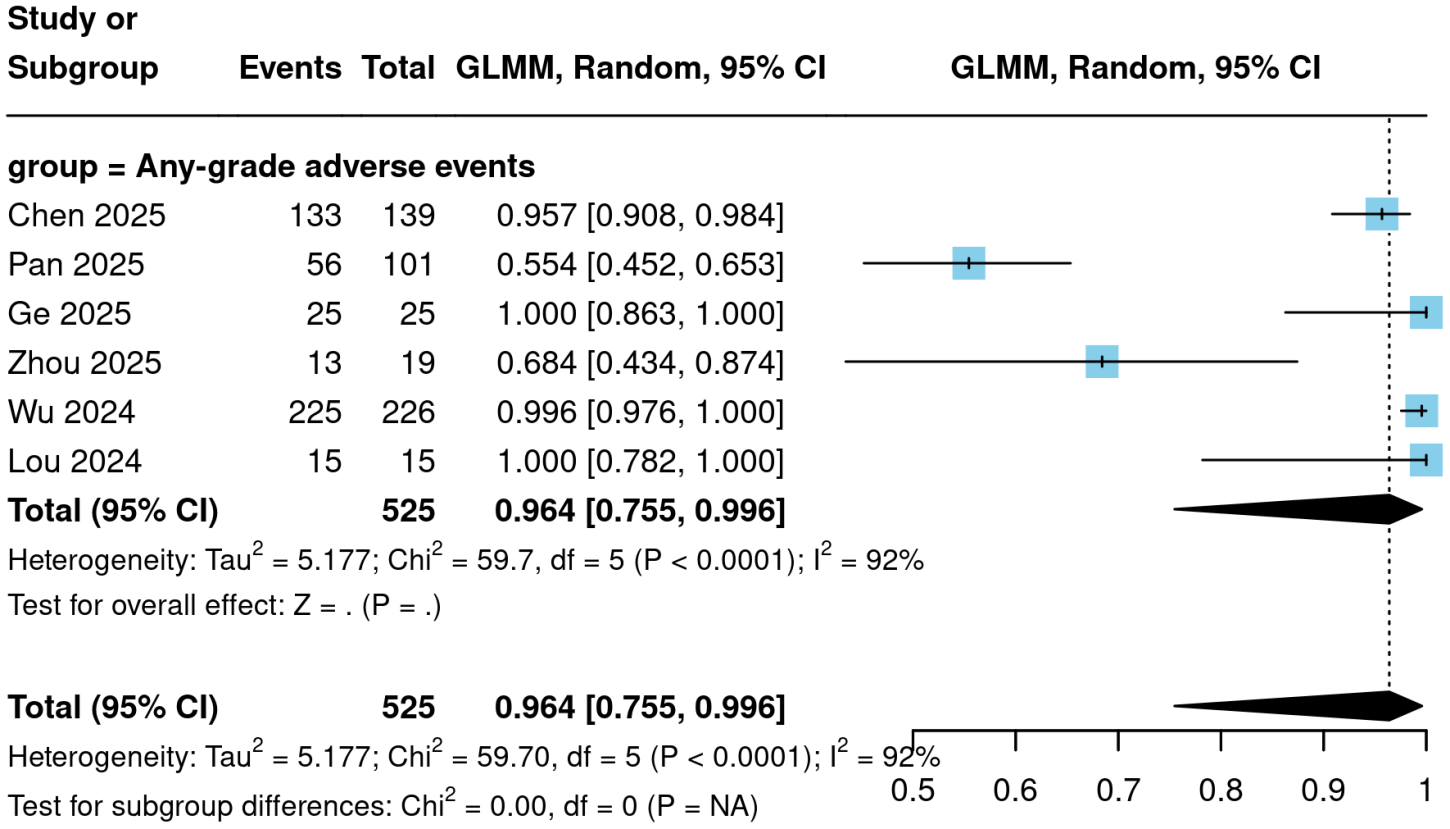
Forest plot of any grade TRAEs.

**Figure 6:**
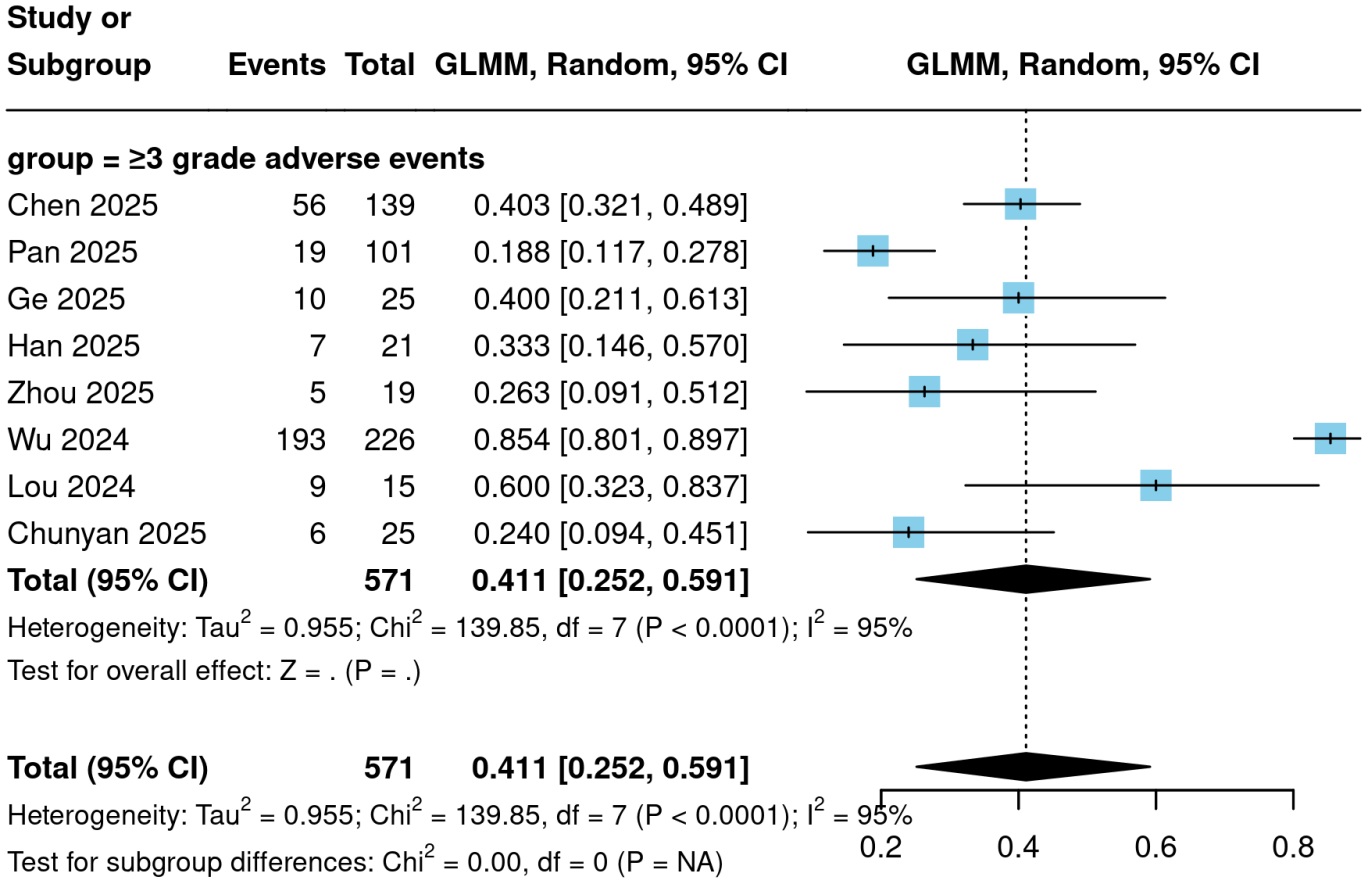
Forest plot of adverse events of grade 3 or above.

**Figure 7:**
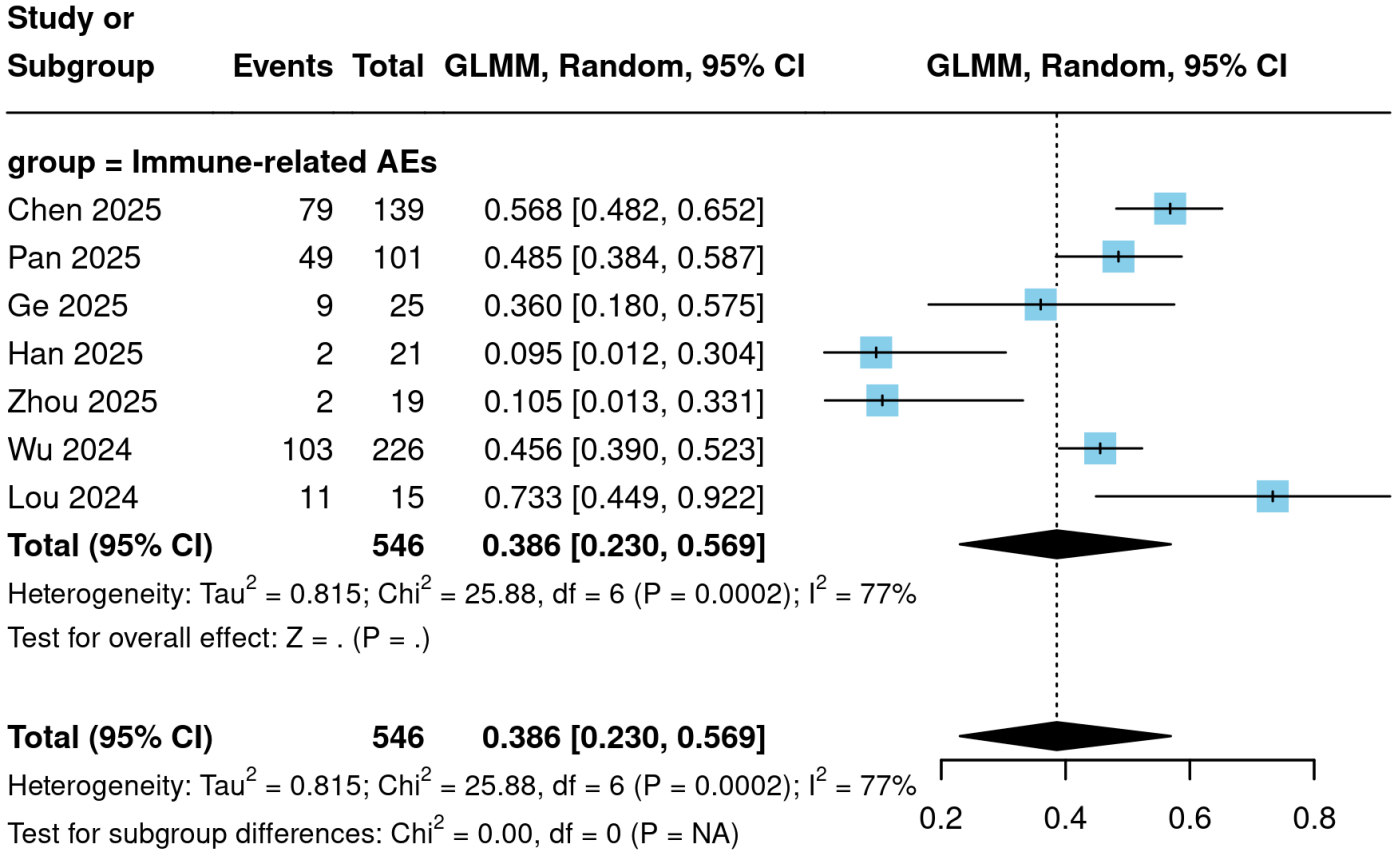
Forest plot of any irAEs.

To further evaluate the safety profile of Cadonilimab, the incidence of various adverse events was analyzed. The meta-analysis showed that the incidence of treatment-related adverse events (TRAEs) of any grade was 96.4% (95% CI = 75.5%-99.6%; **Figure 5**). The incidence of grade ≥3 TRAEs was 41.1% (95% CI = 25.2%-59.1%; **Figure 6**). The incidence of immune-related adverse events (irAEs) was 38.6% (95% CI = 23.0%-56.9%; **Figure 7**). Other specific categories of adverse events included thrombocytopenia, neutropenia, leukopenia, anemia, elevated ALT, elevated AST, hypoalbuminemia, nausea/vomiting, diarrhea, hypothyroidism, and hyperthyroidism. The detailed pooled results from the meta-analysis are presented in **Table 2**.

**Table 2:** Summary of adverse reaction results for specific categories.

Outcomes		No. of studies	Ratio (95% CI)	Heterogeneity	
				I^2^ (%)	P
Hematological toxicity	Thrombocytopenia	10	18.4%(10.6%-30.0%)	87	<0.0001
	Neutropenia	7	26.7%(14.4%-44.1%)	94	<0.0001
	Leukocytopenia	9	47.4%(34.7%-60.5%)	89	<0.0001
	Anemia	10	54.9%(39.3%-69.6%)	92	<0.0001
Hepatic toxicity	ALT elevation	7	23.0%(19.1%-27.4%)	37	0.1493
	AST elevation	7	23.1%(19.4%-27.2%)	24	0.2448
	Hypoalbuminemia	4	26.2%(7.6%-60.4%)	97	<0.0001
Gastrointestinal toxicity	Nausea/Vomiting	6	24.9%(5.6%-65.1%)	97	<0.0001
	Diarrhea	5	14.6%(6.3%-30.2%)	92	<0.0001
Endocrine system toxicity	Hypothyroidism	9	23.4%(18.3%-29.3%)	51	0.0396
	Hyperthyroidism	6	9.3%(6.0%-14.1%)	34	0.1830

### 3.5 Prognostic assessments

**Figure 8:**
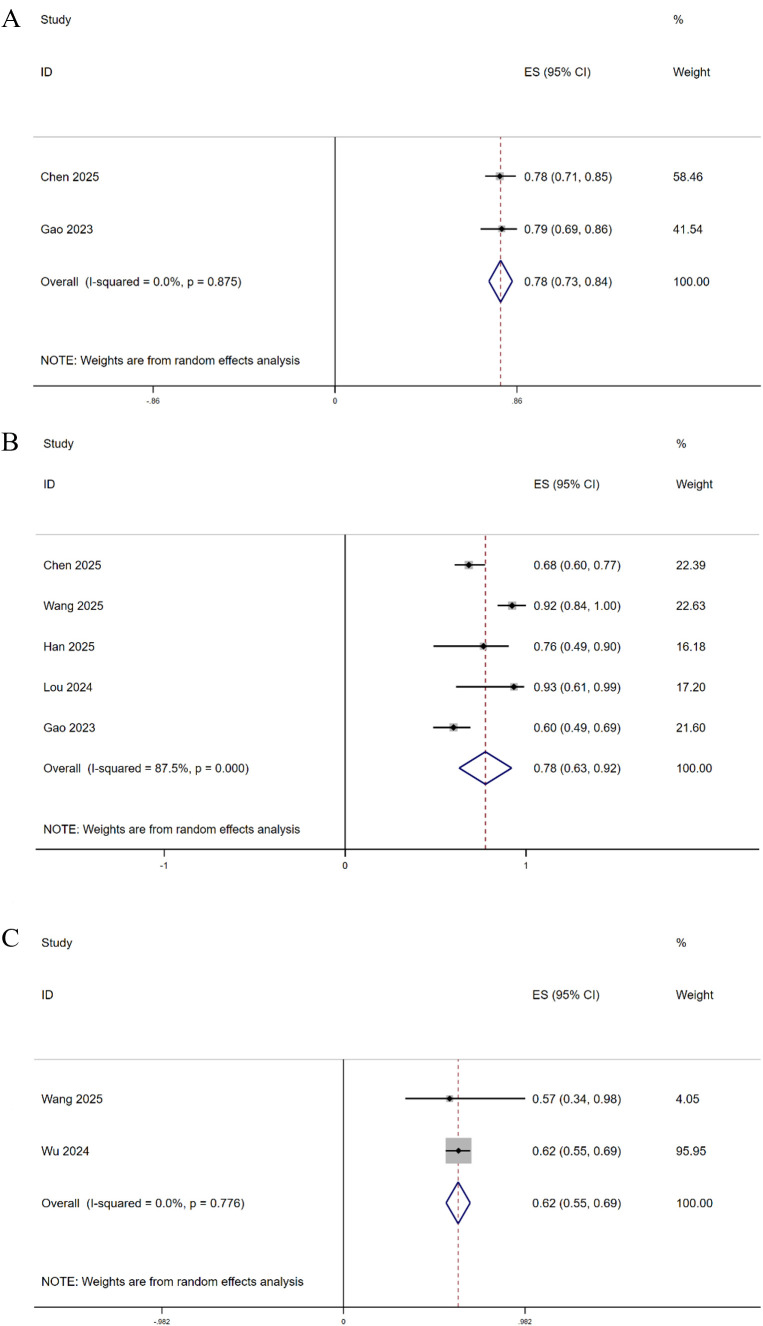
Forest plots of 6-month OS **(A)**, 12-month OS **(B)**, and 24-month OS **(C)**.

Six studies reported overall survival (OS) data. Among these, two studies reported the 6-month OS rate, five studies reported the 12-month OS rate, and two studies reported the 24-month OS rate. The pooled estimates were 78%, 78%, and 62%, respectively (**Figure 8**).

**Figure 9:**
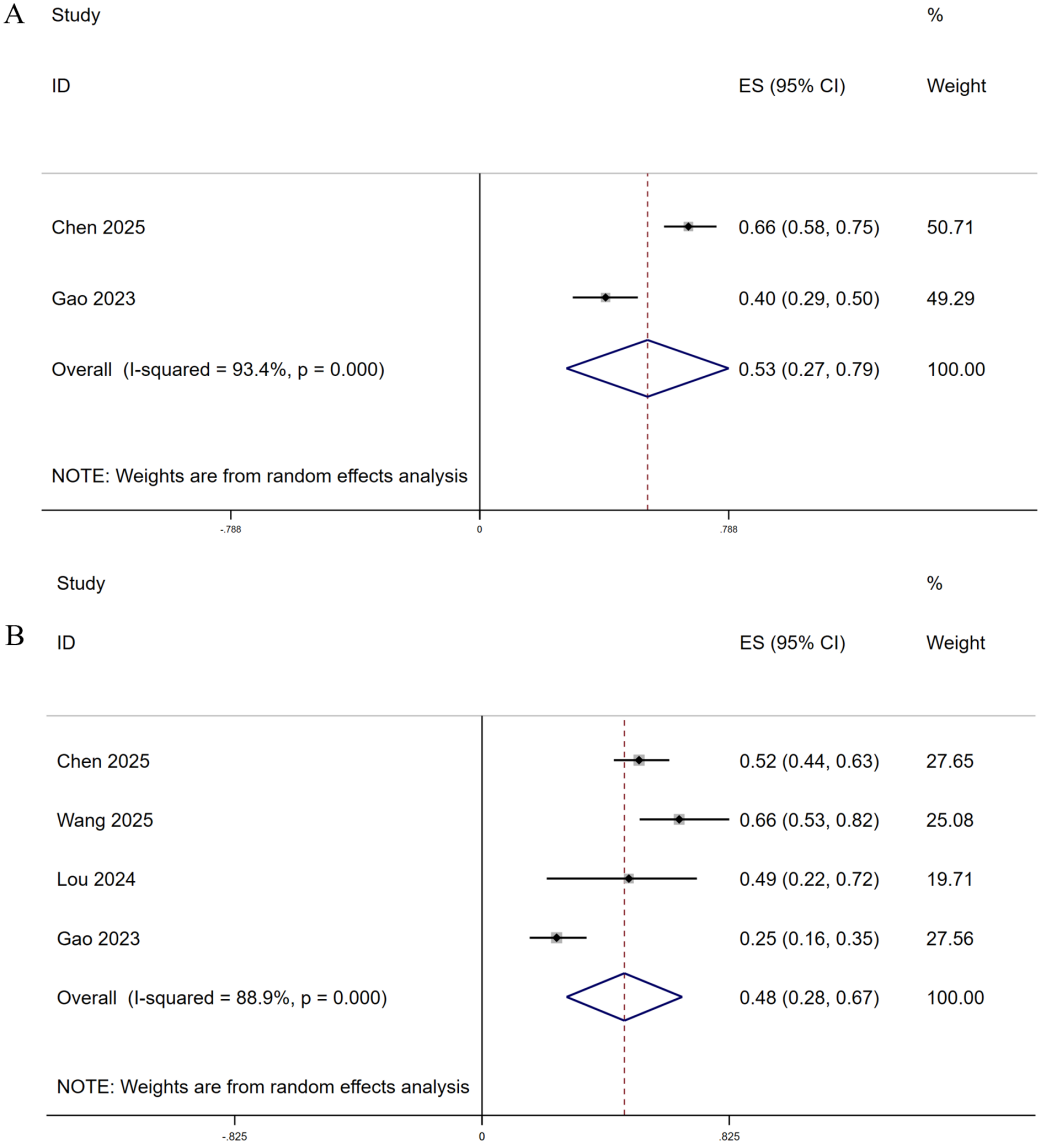
Forest plots of 6-month PFS **(A)** and 12-month PFS **(B)**.

Four studies reported progression-free survival (PFS) data. Of these, two studies reported the 6-month PFS rate and four studies reported the 12-month PFS rate. The pooled estimates were 53% and 48%, respectively (**Figure 9**).

### 3.6 Subgroup analysis

Given the presence of heterogeneity, we performed subgroup analyses based on study design, geographical scope, sample size, and dosage regimen to explore potential sources of heterogeneity, influencing factors, and their potential impact on the results. The results of the subgroup analyses are presented in **Table 3**.

**Table 3:** Summary of subgroup analysis results.

Subgroup		Outcomes	No. of studies	Ratio (95% CI)	Heterogeneity	
					I^2^ (%)	P
Study design	Clinical trial	ORR	3	62.6%(34.2%-84.3%)	97	<0.0001
		DCR	3	90.0%(48.9%-98.8%)	97	<0.0001
		≥3 grade AEs	2	78.9%(58.4%-90.9%)	83	0.0151
	Retrospective cohort study	ORR	7	55.5%(42.3%-68.0%)	79	<0.0001
		DCR	7	79.2%(67.8%-87.4%)	76	0.0004
		≥3 grade AEs	6	29.9%(22.2%-39.0%)	64	0.0167
Research scale	Multicenter	ORR	3	53.3%(26.0%-78.7%)	98	<0.0001
		DCR	3	77.5%(49.8%-92.3%)	97	<0.0001
		≥3 grade AEs	2	66.6%(30.6%-90.0%)	99	<0.0001
	Single-center	ORR	7	60.0%(48.0%-70.9%)	59	0.0221
		DCR	7	83.6%(70.2%-91.7%)	71	0.0018
		≥3 grade AEs	6	30.6%(21.1%-42.2%)	61	0.0238
Sample size	>100	ORR	4	54.9%(33.3%-74.8%)	97	<0.0001
		DCR	4	80.3%(60.3%-91.6%)	96	<0.0001
		≥3 grade AEs	3	49.4%(17.4%-81.8%)	98	<0.0001
	<100	ORR	6	60.2%(44.8%-73.8%)	66	0.0114
		DCR	6	83.1%(65.6%-92.7%)	71	0.0041
		≥3 grade AEs	5	35.3%(26.2%-45.5%)	33	0.2009
Cadonilimab dosage	10mg/kg, q3w	ORR	7	64.0%(49.1%-76.5%)	86	<0.0001
		DCR	7	83.8%(71.8%-91.3%)	83	<0.0001
		≥3 grade AEs	6	38.2%(19.5%-61.1%)	96	<0.0001
	Others	ORR	3	37.9%(32.0%-44.1%)	67	0.0489
		DCR	3	79.6%(37.4%-96.2%)	80	0.0068
		≥3 grade AEs	2	42.2%(34.7%-50.1%)	52	0.1498

### 3.7 Sensitivity analysis

**Figure 10:**
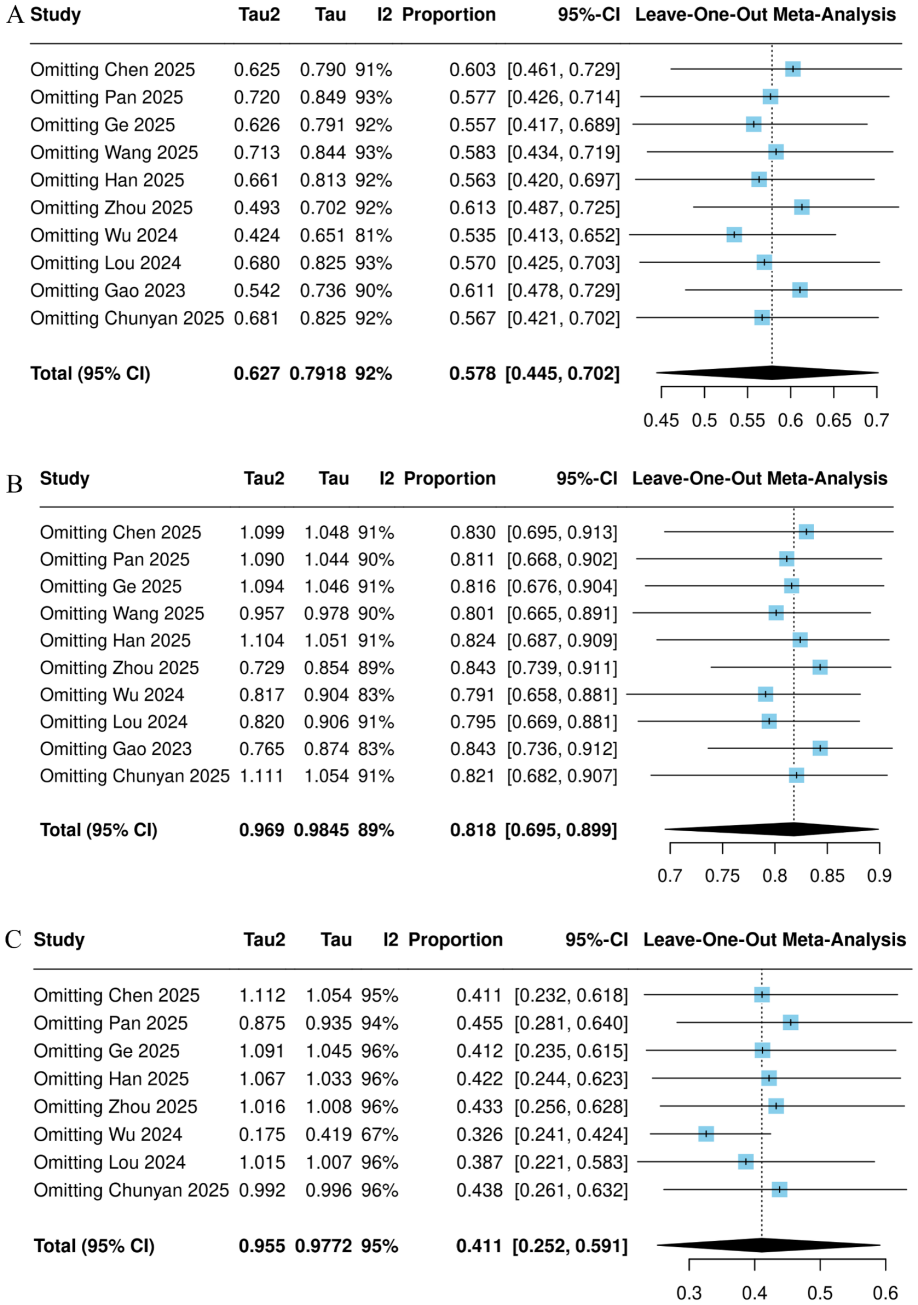
Sensitivity analysis plots of ORR **(A)**, DCR **(B)**, and adverse events of grade 3 or above **(C)**.

A sensitivity analysis was conducted by sequentially excluding individual studies for the outcomes of ORR, DCR, and the incidence of grade ≥3 adverse events. The results indicated that after excluding studies such as Wu 2024, the heterogeneity for ORR decreased (I² = 81%), and the pooled result also decreased to 53.5% (95% CI = 41.3%-65.2%). After excluding studies such as Gao 2023, the heterogeneity for DCR decreased (I² = 83%), and the pooled result increased to 84.3% (95% CI = 73.6%-91.2%). Following the exclusion of studies such as Wu 2024, the heterogeneity for the incidence of grade ≥3 adverse events decreased (I² = 67%), and the pooled result also decreased to 32.6% (95% CI = 24.1%-42.4%). These results are shown in **Figure 10**.

### 3.8 Risk of bias assessments

**Figure 11:**
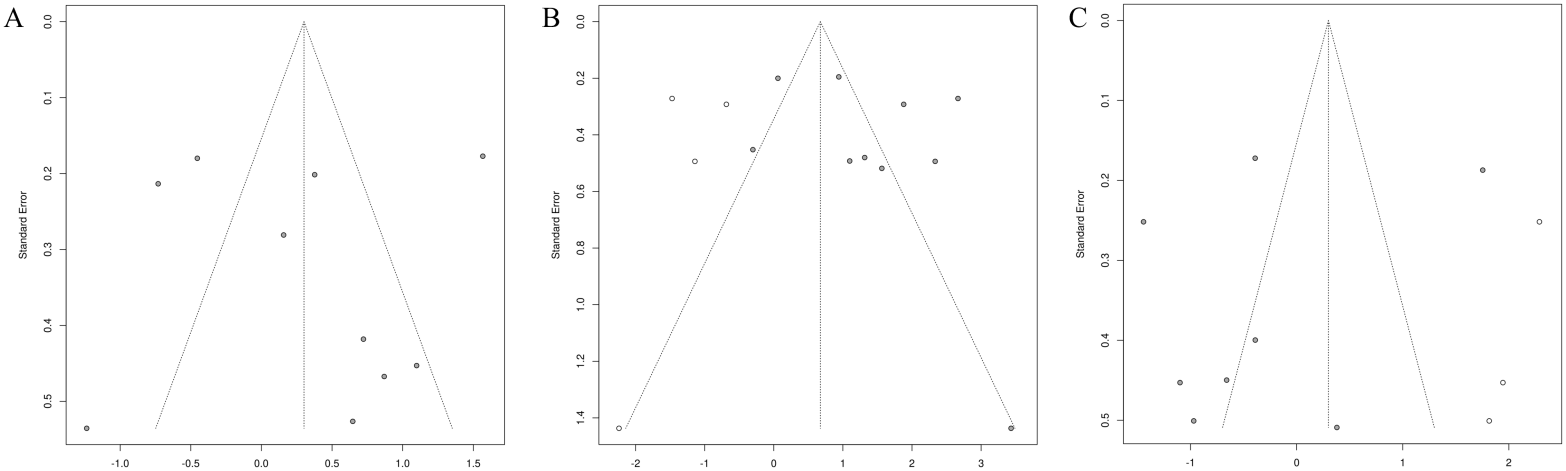
Funnel plots of ORR **(A)**, DCR **(B)**, and adverse events of grade 3 or above **(C)**.

Funnel plots were constructed for the three outcome measures (ORR, DCR, and grade ≥3 adverse events). Visual inspection indicated general symmetry, suggesting no significant publication bias (**Figure 11**). Egger’s test was further performed on these outcomes, with all results showing P > 0.05 (ORR: P = 0.9902; DCR: P = 0.3636; grade ≥3 adverse events: P = 0.3906). This suggests a low likelihood of significant publication bias. However, given the relatively small number of studies included in each analysis, this result should be interpreted with caution.

## 4 Discussion

Historically, patients with metastatic disease lacked a unified treatment strategy due to the heterogeneity of clinical presentations, with a median overall survival of only 8 to 13 months (24). The treatment landscape has undergone a significant transformation in recent years. According to the NCCN Guidelines Version 2.2026, pembrolizumab or atezolizumab combined with chemotherapy (± bevacizumab) is now the preferred first-line regimen for persistent, recurrent, or metastatic CC. However, despite these combination regimens improving survival outcomes, a subset of patients remains refractory to or progresses on existing therapies, necessitating the continued exploration of novel and effective treatment strategies. Cadonilimab, a bispecific antibody targeting both PD-1 and CTLA-4, has completed two Phase II clinical trials (NCT04380805, NCT04868708) in patients with metastatic or recurrent CC, showing potential for substantial clinical benefit (11, 25).The pivotal phase III clinical trial (COMPASSION-16) of Cadonilimab in advanced CC has been completed and published, establishing its role in first-line therapy in combination with chemotherapy (20). In this context, this meta-analysis aims to integrate all available clinical data, including this phase III trial, to provide a more comprehensive evidence synthesis: to validate the consistency of efficacy across different study designs, from early exploratory to confirmatory trials; to further explore potential differences in efficacy profiles between real-world settings and clinical trials; and finally, to enable a more precise assessment of the safety profile, particularly regarding adverse events, through pooled data. Therefore, this meta-analysis was conducted to systematically evaluate the efficacy and safety of Cadonilimab in patients with recurrent, metastatic, or advanced CC, thereby providing robust evidence-based support for clinical practice. To comprehensively and timely evaluate its efficacy and safety at the current stage, we decided to incorporate all available evidence, although combining different study designs may introduce potential bias. This integrative strategy, despite its limitations, holds distinct value: RCT data provide verification of efficacy under ideal controlled conditions, while real-world data complement this by offering information on its applicability in complex clinical settings. Subsequent subgroup and sensitivity analyses were performed to address these aspects, thereby providing a continuous spectrum of evidence for clinical decision-making, ranging from ideal efficacy to real-world effectiveness.

This systematic review synthesized evidence from real-world studies and clinical trials investigating Cadonilimab for metastatic, recurrent, and advanced CC, encompassing 10 studies with 728 participants. Utilizing single-arm proportion meta-analysis, data from eligible treatment arms in comparative studies were extracted and analyzed. The findings demonstrate promising efficacy: the pooled ORR for Cadonilimab in recurrent, metastatic, or advanced CC was 57.8%, with a DCR of 81.8% and a PD rate of only 18.2%. These results highlight the considerable potential of Cadonilimab in managing advanced disease, indicating significant advantages in tumor control and progression delay. For context, the KEYNOTE-158 phase II basket study reported an ORR of only 12.2% with Pembrolizumab in previously treated advanced CC (NCT02628067) (26), while the CheckMate 358 phase I/II trial reported an ORR of 26.3% with Nivolumab monotherapy for recurrent or metastatic CC (NCT02488759) (27). In contrast, combination therapy with PD-1 and CTLA-4 inhibitors, such as the specific regimen of nivolumab plus ipilimumab, has elevated the ORR to 41.3%. This comparison highlights the potential of bispecific antibodies. It suggests that although previously used single-agent immune checkpoint inhibitors have demonstrated certain efficacy, their effectiveness remains relatively limited. Furthermore, a notably high rate of primary resistance has been observed in clinical practice. One possible mechanism for this resistance is immune escape occurring in both the initiation and effector phases of the immune response (28, 29). In recent years, the treatment landscape for recurrent/metastatic CC has been fundamentally transformed by three pivotal phase III randomized controlled trials (BEATcc, KEYNOTE-826, and COMPASSION-16), which established immune checkpoint inhibitor plus chemotherapy (± bevacizumab) as the new first-line standard of care (20, 30, 31). To further enhance efficacy, additional combination therapies involving PD-1 and CTLA-4 inhibitors are being explored (28). Against this background, a systematic evaluation of Cadonilimab, as a novel bispecific antibody, across different levels of evidence is warranted. Mechanistically, Cadonilimab blocks the interactions of PD-1 with PD-L1/PD-L2 and CTLA-4 with B7-1/B7-2, while minimizing Fc-mediated effector functions like antibody-dependent cellular cytotoxicity and cytokine release (11). Bispecific antibodies, capable of recognizing two distinct antigens or epitopes, often demonstrate superior clinical efficacy compared to monoclonal antibodies (32). Compared to conventional anti-PD-1 and anti-CTLA-4 antibodies, Cadonilimab may exhibit preferential retention in tumor tissue. Its tetravalent structure enhances target specificity and immune synapse formation, potentially reducing off-target effects and contributing to the improved efficacy and safety profile observed (33, 34). Importantly, indirect comparisons between Cadonilimab and other ICIs are limited by differences in trial designs, patient cohorts, and clinical settings, and should therefore be interpreted with caution. Regarding prognosis, the 12-month OS rate and PFS rate were 78% and 48%, respectively, suggesting that intervention with this drug may be beneficial in improving patient survival. However, only a limited number of studies reported these survival outcomes, which may reduce statistical power, and caution is warranted when interpreting the long-term efficacy.

Regarding safety, it is noteworthy that the pooled analysis indicated that the majority of patients experienced adverse events of any grade, with the incidence of grade ≥3 adverse events being 41.1% and that of irAEs being 38.6%. The mechanism of action of immunotherapy inevitably leads to certain adverse events, affecting various organs or systems throughout the body. The spectrum of reported adverse events includes thrombocytopenia, neutropenia, leukopenia, anemia, elevated ALT, elevated AST, hypoalbuminemia, nausea/vomiting, diarrhea, hypothyroidism, and hyperthyroidism. These occurrences may be influenced by factors such as the patient’s baseline condition and individual characteristics, drug dosage and treatment duration, and the status of the tumor microenvironment. Furthermore, the use of Cadonilimab is not recommended in patients with moderate or severe hepatic impairment or severe renal impairment (11). irAEs represent a critical concern requiring meticulous management and monitoring throughout the treatment course. There is a need to identify more reliable biomarkers to predict patient responses and to develop strategies for anticipating and managing various irAEs (35). Depending on the severity of the reaction, treatment interruption and administration of corticosteroids are necessary until the immune-related adverse event improves to grade ≤1. If the irAEs continue to worsen or shows no improvement, non-corticosteroid immunosuppressive therapy should be considered (11).

Given the observed heterogeneity, we performed subgroup analyses by study design, geographical scope, sample size, and dosage regimen to explore potential sources of variation. Notably, the pooled ORR and DCR in clinical trials were 62.6% and 90.0%, respectively, whereas real-world studies demonstrated lower rates (ORR: 55.5%; DCR: 79.2%). This suggests that real-world populations likely encompass a broader spectrum of clinical scenarios, including complex cases typically excluded from clinical trials. These patients may present with poorer baseline conditions and more comorbidities, potentially contributing to reduced response rates. Regarding dosage, the 10 mg/kg every 3 weeks (q3w) regimen was associated with higher ORR and DCR compared to other dosing schedules. In September 2024, China’s National Medical Products Administration approved Cadonilimab at 10 mg/kg q3w in combination with fluoropyrimidine and platinum-based chemotherapy for the first-line treatment of locally advanced unresectable or metastatic gastric or gastroesophageal junction (G/GEJ) adenocarcinoma (36). This dosing regimen may also demonstrate favorable efficacy in patients with recurrent or metastatic CC. Furthermore, subgroup analyses indicated that studies with sample sizes < 100 reported higher ORR (60.2% vs. 54.9%) and lower incidence of TRAEs (35.3% vs. 49.4%) compared to those with larger sample sizes. Multicenter studies showed lower ORR (53.3% vs. 60.0%) and higher TRAEs incidence (66.6% vs. 30.6%) than single-center studies. Heterogeneity was reduced to varying degrees across all stratified subgroups following these analyses.

It is noteworthy that the pooled subgroup results from small-sample, single-center studies reported higher ORR and a lower incidence of TRAEs. This phenomenon may be attributed to stricter practical application of patient enrollment criteria in these studies, where investigators might have preferentially selected patients with better performance status, fewer comorbidities, relatively lower tumor burden, or those anticipated to have a better response to immunotherapy. Such potential selection bias could inflate the observed ORR and lead to a lower reported incidence of TRAEs due to a patient population with more favorable baseline conditions. In contrast, large-sample, multicenter studies aim to reflect a broader patient population by including more cases with unfavorable prognostic factors and greater overall representativeness, making their results more aligned with real-world effectiveness.

In clinical practice, the potential sources of heterogeneity are far more complex than what statistical models can capture. Variations in PD-L1 expression status, lines of prior therapy, individual patient characteristics, differences in treatment protocols and management norms across centers, dose adjustment strategies, and experience in managing adverse reactions are all potential clinical sources of heterogeneity. When referencing clinical trial data to formulate treatment decisions for patients, clinicians must exercise greater caution. They should clearly determine PD-L1 status, consider prior treatment history, and integrate real-world evidence to develop a more realistic assessment of the expected therapeutic benefit for the individual patient.

This study possesses several strengths. First, to our knowledge, it represents the first meta-analysis to systematically evaluate the safety and efficacy of Cadonilimab specifically in recurrent, metastatic, and advanced CC, thereby providing a novel synthesis of existing evidence. Second, the inclusion of real-world study data enhances the generalizability of the findings to the complex patient populations encountered in routine clinical practice. Third, all included studies were published within the last three years, ensuring that the integrated evidence accurately reflects the most recent clinical advancements. However, this study also has limitations. First, considerable heterogeneity was observed across most analyses. Although subgroup and sensitivity analyses were performed, the source of residual heterogeneity for some outcomes remains unexplained, necessitating a cautious interpretation of the pooled results. Second, As the drug was initially developed by a Chinese company and its clinical trials were conducted predominantly in China, all eligible studies included in this meta-analysis originated from China. Consequently, the findings may be more applicable to the Chinese patient population, as results may vary across different regions and ethnic groups. Therefore, future multi-regional, multi-center studies are warranted to validate the generalizability of these findings. Third, limited by the number and quality of the included studies, the OS and PFS results derived from the available data are subject to considerable uncertainty. Their clinical significance should be considered exploratory, and caution is warranted in their interpretation. The assessed survival benefits should not be regarded as conclusive evidence. Fourth, a meta-analysis of single-arm data is inherently descriptive rather than comparative. Although the pooled results indicate promising anti-tumor activity for Cadonilimab, they do not allow for direct inference regarding its superiority, equivalence, or non-inferiority to existing standard treatments such as PD-1 monotherapy or combination with chemotherapy. The level of evidence is limited, and some unknown biases may exist. Future research should prioritize randomized controlled trials comparing Cadonilimab with other bispecific antibodies, to comprehensively evaluate and compare different therapeutic strategies and better inform clinical decision-making.

## 5 Conclusion

In summary, this study systematically evaluated the safety and efficacy of Cadonilimab in patients with recurrent, metastatic, or advanced CC. Based on the currently available evidence predominantly from single-arm and retrospective studies, this systematic review and meta-analysis suggests that Cadonilimab is associated with favorable antitumor activity and a manageable safety profile in patients with recurrent, metastatic, or advanced CC. Hints of a potential survival benefit were observed, but this estimate is based on limited data and requires cautious interpretation. During treatment, close monitoring for irAEs is essential, with prompt pharmacological intervention upon detection to mitigate risks while striving for optimal therapeutic outcomes. Future large-scale, multi-regional randomized controlled trials are warranted to explore superior treatment strategies for advanced CC, elevate the level of evidence-based medicine, and better inform clinical decision-making.

## Data Availability

Publicly available datasets were analyzed in this study. This data can be found here: The original data utilized in this systematic review and meta-analysis were derived from previously published studies. All data generated or analyzed during this study are included in this published article (and its supplementary information files), and are also available from the corresponding studies cited in the reference list.
