# Supplementary material for: Efficacy and safety of Cadonilimab in the treatment of recurrent/metastatic and advanced cervical cancer: a systematic review and meta-analysis
Source: Front Immunol. 2026 Jan 12;16:1729380. doi: 10.3389/fimmu.2025.1729380 (PMC12832733; doi:10.3389/fimmu.2025.1729380)
Supplement: Supplementary file 1 [file DataSheet1.docx]

(Cadonilimab[Title/Abstract]) AND (((((((((((((((((((((((((Uterine Cervical Neoplasms[MeSH Terms])) OR (Cervical Neoplasm, Uterine[Title/Abstract])) OR (Neoplasm, Uterine Cervical[Title/Abstract])) OR (Uterine Cervical Neoplasm[Title/Abstract])) OR (Neoplasms, Cervix[Title/Abstract])) OR (Cervix Neoplasm[Title/Abstract])) OR (Neoplasm, Cervix[Title/Abstract])) OR (Cervix Neoplasms[Title/Abstract])) OR (Cervical Neoplasms[Title/Abstract])) OR (Cervical Neoplasm[Title/Abstract])) OR (Neoplasms, Cervical[Title/Abstract])) OR (Cancer of the Uterine Cervix[Title/Abstract])) OR (Cancer of Cervix[Title/Abstract])) OR (Cancer of the Cervix[Title/Abstract])) OR (Cervix Cancer[Title/Abstract])) OR (Cancer, Cervix[Title/Abstract])) OR (Uterine Cervical Cancer[Title/Abstract])) OR (Cancer, Uterine Cervical[Title/Abstract])) OR (Cervical Cancer, Uterine[Title/Abstract])) OR (Uterine Cervical Cancers[Title/Abstract])) OR (Cervical Cancer[Title/Abstract])) OR (Cancer, Cervical[Title/Abstract])) OR (Cervical Cancers[Title/Abstract])))
